# Cerebral vasculitis and intracranial multiple aneurysms in a child with Lyme neuroborreliosis

**DOI:** 10.1099/jmmcr.0.005090

**Published:** 2017-04-21

**Authors:** Elisa Kortela, Jukka Hytönen, Jussi Numminen, Margit Overmyer, Harri Saxen, Jarmo Oksi

**Affiliations:** ^1^​ Division of Infectious Diseases, Faculty of Medicine, University of Turku, University of Helsinki, Helsinki University Hospital, P.O. Box 348, 00029 HUS, Finland; ^2^​ Department of Medical Microbiology and Immunology and Microbiology and Genetics Department, University of Turku, Turku University Hospital, Turku, Finland; ^3^​ Helsinki Medical Imaging Centre, University of Helsinki, Helsinki, Finland; ^4^​ Children's Hospital, Helsinki University Hospital, Helsinki, Finland; ^5^​ Children's Hospital, University of Helsinki and Helsinki University Hospital, Helsinki, Finland; ^6^​ Department of Infectious Diseases, Division of Medicine, Faculty of Medicine, University of Turku, Turku University Hospital, P.O. Box 52, 20521 Turku, Finland

**Keywords:** lyme disease, *Borrelia burgdorferi*, meningoencephalitis, cerebral vasculitis, ischemic stroke, aneurysms

## Abstract

**Introduction.** Lyme borreliosis is a multisystem tick-borne disease caused by *Borrelia burgdo* *rferi*. Neurological manifestations are reported in up to 15 % of adult patients with Lyme disease, while the frequency among children is higher. The most common manifestations are painful radiculopathy, facial nerve paresis and lymphocytic meningitis. Epileptic seizures and cerebral vasculitis with stroke or aneurysms are very rare complications.

**Case presentation.** We describe a paediatric patient with sensorineural auditory dysfunction, headache, fatigue and epileptic seizures as sequelae of meningoencephalitis/Lyme neuroborreliosis (LNB) caused by *B. burgdo* *rferi*. Brain magnetic resonance imaging revealed widespread enhancement of the leptomeninges, cranial nerves and artery walls compatible with vasculitis and disturbances in cerebrospinal fluid (CSF) circulation. The patient was treated with ceftriaxone for 2 weeks. Two years later, the patient had an ischemic stroke. Brain magnetic resonance angiography revealed multiple aneurysms, which were not present previously. The largest aneurysm was operated rapidly. The patient was treated with another course of intravenous ceftriaxone for 4 weeks and pulse therapy with corticosteroids. He recovered well.

**Conclusion:**

.**** This unique case demonstrates complications of LNB that can result in serious morbidity or even mortality. Lumbar puncture and analysis should be considered for paediatric patients with epileptic seizures or cerebrovascular events living in a Lyme borreliosis endemic area.

## Abbreviations

CNS, central nervous system; CSF, cerebrospinal fluid; CTA, computer tomography angiography; EEG, electroencephalography; LNB, Lyme neuroborreliosis; MRA, magnetic resonance angiography; MRI, magnetic resonance imaging.

## Introduction

Lyme borreliosis is a multisystem tick-borne disease caused by the spirochete *Borrelia burgdorferi sensu lato*. Nervous system involvement in Lyme borreliosis occurs in up to 15 % of adult patients, and the most common manifestations of Lyme neuroborreliosis (LNB) in Europe are painful radiculopathy, lymphocytic meningitis and facial nerve paresis. Cerebral vasculitis seems to be a rare complication of Lyme borreliosis, estimated to affect about 0.3 % of all patients in endemic areas [1, 2]. The most common symptom of cerebral vasculitis is headache. Other typical manifestations are cognitive impairment, disturbance of gait and focal neurological deficit or stroke. Ataxia, seizure and diplopia are less frequent symptoms [3, 4].

Diagnosis of LNB relies on the demonstration of symptoms and signs compatible with the disease, and on the demonstration of *B. burgdorferi-*specific intrathecal antibody production [positive cerebrospinal fluid (CSF)/serum antibody index]. Unfortunately, the antibodies may remain elevated for years after antibiotic treatment of LNB, which hampers the diagnostics of a reinfection. The measurement of the concentration of a chemokine called CXCL13 in the CSF of LNB patients has appeared during recent years as an additional laboratory test that can be used to differentiate an acute case of LNB from a previously treated one [5]. CXCL13 concentrations above 250–415 pg ml^−1^, in combination with positive borrelia serology, are suggestive of an acute infection [6, 7].

In this case report, we describe a rare case with stroke and aneurysms in a child due to cerebral vasculitis caused by LNB. We have previously published a case report of three LNB patients with cerebral vasculitis and intracranial aneurysms [8]. In the literature, no other case reports on the association of LNB and intracranial aneurysms have been published, to the best of our knowledge. However, cerebral vasculitis and/or stroke have been described in several patients with LNB [4, 9].

## Case Report

An 8-year-old boy, who lived at that time in another country, had been experiencing attacks with intense headache and fever for a few months. We had no medical records from that period, but according to the patient′s mother, a brain magnetic resonance imaging (MRI) scan that was performed was normal. The attacks with headache and fever resolved spontaneously. However, the patient had slight motor clumsiness, and complained of fatigue and learning difficulty in school. Three years later, he received hearing aids because of sensorineural auditory dysfunction in both ears. Otherwise, he had been healthy.

**Fig. 1.**
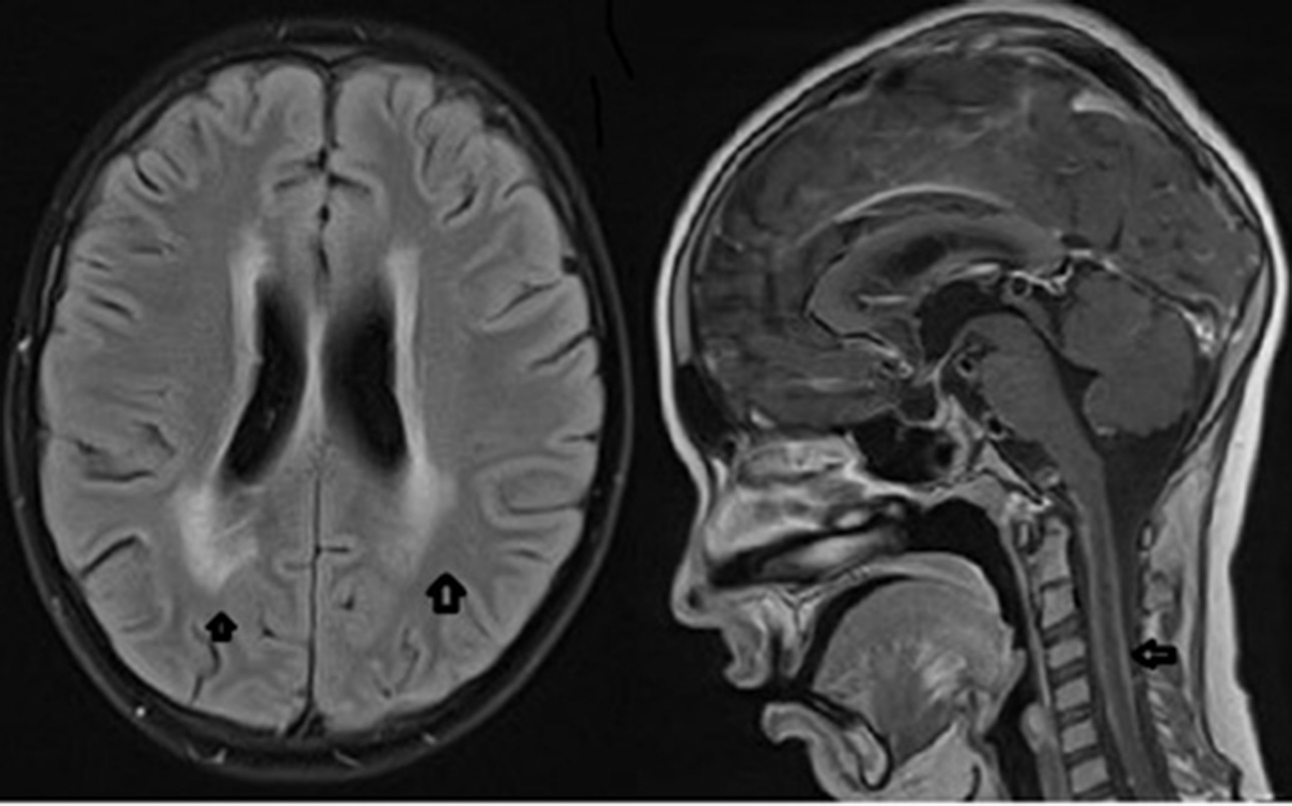
At the age of 12 years, after the first seizure with hallucination, vomiting and aphasia, brain MRI with fluid attenuation inversion recovery (FLAIR) (left) sequence revealed high intensity foci (indicated by arrows), and contrast-enhanced T1 sequence (right) revealed leptomeningeal and perivascular enhancement reflecting inflammation (indicated by an arrow).

At the age of 12 years, the patient had a seizure with hallucination, vomiting and aphasia. Brain MRI revealed multiple findings with leptomeningeal, cranial nerve and artery wall enhancement compatible with vasculitis and disturbances in CSF circulation (Fig. 1). The changes were considered to be inflammatory, but it could not be judged whether they were acute or chronic. Electroencephalography (EEG) showed focal left-sided slow spike-wave discharges, which were regarded as an epileptiformic abnormality. Anticonvulsive medication with oxcarbazepine (14 mg kg^−1^ daily) was instituted. The patient was initially treated in a district hospital but, unfortunately, a CSF analysis was not performed at that time.

During the next 2 months, the patient had two more seizure attacks, and the anticonvulsive medication was changed to valproic acid (18.75 mg kg^−1^ daily). Two months after the first seizure, the patient was admitted again to the hospital because of a fourth seizure. Neurological examination was normal. Brain MRI and magnetic resonance angiography (MRA) showed decreasing intensity of the previous multiple inflammatory findings. No aneurysms were found. CSF analysis showed lymphocytic meningitis (470 leukocytes mm^−3^, 99 % lymphocytes) and a high level of protein (4390 mg l^−1^).

Because of the lymphocytic meningitis and seizures, the patient was transferred to the Helsinki University Hospital. A few days later, the rest of the results of the CSF analysis were received. The *Borrelia*-specific CSF/serum IgG-antibody index was 4.5 (normal value <0.3) and the IgM-antibody index was 5.8 (normal value <1.5), both clearly positive (Borrelia afzelii + VlsE IgG ELISA IgG test kit and Borrelia afzelii IgM ELISA IgM test kit; Sekisui Virotech). The patient was treated with intravenous ceftriaxone (2 g daily) for 14 days. The anticonvulsive medication was discontinued. After the antibiotic treatment, the auditory dysfunction normalized and the boy did not suffer from any epileptic seizures or attacks. However, the patient still had learning difficulties.

**Fig. 2.**
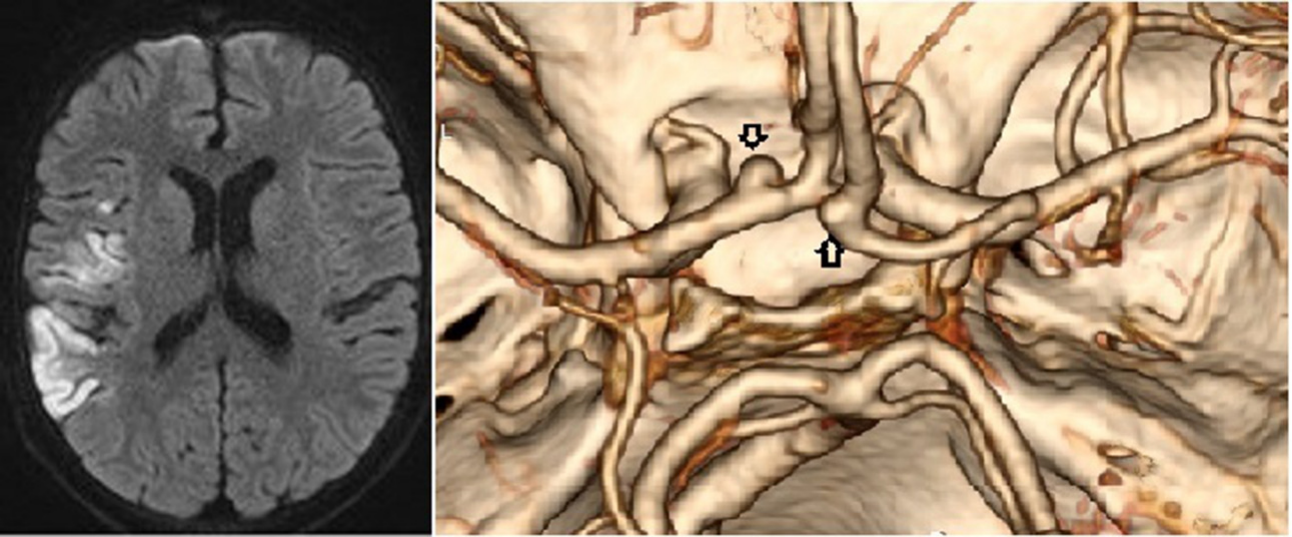
At the age of 14 years, after the stroke, brain CTA (right) showed an aneurysm in the left anterior cerebral artery with a largest diameter of 8 mm and smaller aneurysm of 2 mm in diameter in the right anterior cerebral artery (indicated by arrows). Two other small aneurysms are not presented in this figure. Brain MRI with diffusion weighted sequence (left) demonstrated an acute right temporal infarct.

When he was 14 years old, the patient suddenly presented with left hemiparesis and aphasia. The condition normalized in a few hours. EEG was normal. CSF examination revealed a slightly elevated level of protein (591 mg l^−1^), no leukocytes and a normal level of glucose (Table 1). However, the level of borrelia-specific IgM antibodies was slightly elevated and the IgG antibodies clearly elevated compatible with a previously treated LNB (Table 1). The level of CXCL13 was low (58 pg ml^−1^) and, thus, not suggestive of an ongoing LNB. Brain MRI and MRA demonstrated an acute right temporal infarct. Additionally, in the left anterior cerebral artery, an aneurysm was detected with a largest diameter of 8 mm and partial thrombosis in it. (Fig.2) Two small aneurysms of 2 mm in diameter in the right anterior cerebral artery and in the left medial cerebral artery at the level of the M2/3 junction were also observed. Carotid artery MRA and echocardiography were normal. After 5 days of hospitalization, the patient was discharged with a daily 200 mg dose of acetylsalicylic acid. A surgical clipping of the biggest aneurysm was planned to be performed 3 months later.

**Table 1.** CSF analysis For the whole cell antigen IgM and IgG (in-house assays), the normal value is <25 Enzyme Immunoassay Unit (EIU). For the Lyme index [C6 B. burgdorferi (Lyme) ELISA kit; Immunetics], the normal value is <0.9.

CSF sample	No. of erythrocytes (cells mm^−3^)	No. of leukocytes (cells mm^−3^)	Protein (mg l^−1^)	Whole cell antigen IgM (EIU)	Whole cell antigen IgG (EIU)	Lyme index	CXCL13 (pg ml^−1^)
At the age of 14 years 3 months	82	0	591	4.8	117	7.12	58
The patient was operated on 11 days after the first sample.							
15 days after the first sample	3467	34	2018	20.6	108	8.06	5929
2 months after the first sample	30	6	530	1.6	90.5	1.11	167
5 months after the first sample	40	1	509	0.5	68.4	0.74	<7.8

One week later, the patient experienced intensive headache. Brain computed tomography angiography (CTA) and MRA did not show any haemorrhage, the biggest 8 mm aneurysm was unchanged but three other small aneurysms were discovered. Craniotomy was performed immediately, because of the increased risk of aneurysm rupture and subarachnoid haemorrhage. The partially thrombotic aneurysm of the left anterior cerebral artery was surgically clipped. The immediate recovery was complete, but 3 days after the operation the patient again experienced intense headache and aphasia. However, the condition normalized in a few minutes. Brain computed tomography did not show any new findings. On the next day, lumbar puncture was performed and the CSF analysis revealed 3467 erythrocytes mm^−3^ and 34 leucocytes mm^−3^. The CXCL13 level was 5929 pg ml^−1^. Doxycycline (150 mg daily) and acetylsalicylic acid (200 mg daily) were instituted.

One week later, the patient continued to suffer from fatigue. EEG showed a general disturbance, slow wave activity, abnormal reactivity of rhythmic activity, but no spikes or discharges. Partly because of increased CXCL13 levels and still high CSF *B. burgdorferi*-specific antibody levels, new treatment with daily ceftriaxone (1.5 g intravenously), for 4 weeks, was started instead of the doxycycline. At the same time, the patient was treated with five pulses of intravenous methylprednisolone (1 g daily). The dose of acetylsalicylic acid was reduced to 100 mg daily.

## Investigations

At the age of 12, when the lymphocytic meningitis caused by *B. burgdorferi* was diagnosed, several diagnostic tests were performed. No herpes simplex virus, varicella zoster virus, enterovirus or human herpesvirus 6 were detected by PCR in the CSF. No serum IgM or IgG antibodies against tick-borne encephalitis virus, cytomegalovirus or *Toxoplasma gondii* were found. No CSF IgM or IgG antibodies against cytomegalovirus, *Toxoplasma gondii* or measles virus were detected. There were IgG but not IgM antibodies against human herpesvirus 6, varicella zoster virus and *Mycoplasma pneumoniae* in the serum, indicating previous infections caused by these microbes. Human immunodeficiency virus antigen/antibody test was negative. *Treponema pallidum* haemagglutination assays of the serum and CSF samples were negative.

Abdominal ultrasonography and chest X-ray were normal. No bacteria were detected in cultures of any of the CSF samples. No coagulopathy disorder was found.

The borrelia antibody analyses of the patient at the age of 12 and at the age of 14 were performed in different laboratories using different testing methods. Therefore, the results are not comparable to each other.

## Outcome and follow-up

As a 16-year-old, 2 years after the first operation and antibiotic and corticosteroids treatments, the patient had recovered well. He had no neurological deficits, seizures or headaches. However, he still had some learning difficulties in school. A neuropsychological test was performed for the patient after the first operation due to the headaches and learning difficulty. It showed a moderate level of cognitive performance and revealed special difficulty in verbal functions. Whether this was a consequence of neuroborreliosis or a chance association remains unknown.

The size of the aneurysm at the level of the left M2/M3 segment had increased up to 4 mm, while the other two aneurysms were unchanged. A re-craniotomy and surgical clipping of the M2/M3 aneurysm was performed recently. The two smaller aneurysms were followed up by brain MRA.

## Discussion

In the endemic areas of Europe and North America, the prevalence of Lyme borreliosis is approximately 1 in 1000 individuals, with nervous system involvement in up to 15 % of adults [10–12]. Children are more likely than adults to present with LNB in Europe [13, 14]. One of the highest reported incidence of childhood Lyme meningitis is 26/100 000 in South-West Norway [15]. The most typical presentations of LNB are cranial neuritis, meningoradiculitis and lymphocytic meningitis [16, 17]. Ischemic stroke, aneurysms or intracerebral or subarachnoid haemorrhages due to cerebral vasculitis or epileptic seizures are rare complications of LNB [2, 4, 18, 19].

Ischemic stroke is very rare in children [20]. The International Pediatric Stroke Study showed that inflammatory arteriopathies are one of the most important aetiologies of childhood stroke [21]. Infectious agents can cause direct infection of cerebral arteries or be a potential trigger for inflammatory cerebral arteriopathy [22]. Varicella zoster virus is known to cause vasculopathy by spreading from the arterial adventitia transmurally towards the lumen. Stroke can occur months after primary infection with zoster rash and in the absence of rash or CSF pleocytosis [23–25]. Other pathogens are less commonly associated with ischemic stroke in children. In addition to *B. burgdorferi*, human immunodeficiency virus, parvovirus B19, influenza A, enteroviruses and *M. pneumoniae* have occasionally been associated with cerebral arteriopathy and stroke in children [26–30].

Our patient received intravenous ceftriaxone for 2 weeks after the diagnosis of meningoencephalitis and cerebral vasculitis caused by *B. burgdorferi*. Nevertheless, the patient presented an ischemic stroke 2 years after this. At the time of the stroke, there were no signs of active infection in the CSF analysis. However, the intracranial aneurysms had developed during the 2 years.

*B. burgdorferi* has previously been associated with aneurysm formation in abdominal aorta, coronary artery and intracranial arteries [8, 31, 32]. The underlying mechanism is proposed to be similar to that of the spirochete *Treponema pallidum*. Both *Treponema pallidum* and *B. burgdorferi* present vessel tropism. *Treponema pallidum* penetrates the vessel wall and attracts lymphocytes and plasma cells around the vasa vasorum in the adventitia [31, 33]. Endarteritis in the vasa vasorum of large and medium sized arteries makes the vessel wall susceptible to aneurysm formation [34]. Manifestations of neurosyphilis caused by *Treponema pallidum* can be meningitis, meningeal vasculitis, hydrocephalus, general paresis, dementia and spinal cord damage. *Treponema pallidum* has been observed in the leptomeninges and in the affected leptomeningeal arteries [35]. An alternative hypothesis is that *B. burgdorferi* does not induce aneurysms by the direct infection of the vessel wall. Epitopes in the surface of *B. burgdorferi* are similar to aortic vessel wall proteins. In the case of abdominal aortic aneurysm, this could lead to autoimmunity and destruction of the aortic tissue via molecular mimicry [31].

Unruptured intracranial aneurysms are prevalent in 3 % of the adult population [36]. Evidence-based guidelines are established for the care of patients presenting with unruptured intracranial aneurysms. [37, 38] It is suggested that unruptured intracranial aneurysms should be monitored with CTA or MRA annually for 2 to 3 years and every 2 to 5 years after that if the aneurysm is stable [39]. Aneurysm growth over time is believed to be a risk factor for haemorrhage [40, 41]. Patients with intracranial aneurysms should avoid smoking and hypertension [42–44].

Sensorineural hearing loss has been associated with LNB in European patients [45]. Our patient′s auditory dysfunction was diagnosed 1 year before the first epileptic seizure. Brain MRI demonstrated abnormal enhancement of multiple cranial nerves, including the vestibulocochlear nerve (VIII) on both sides. After treatment with antibiotics the hearing loss improved, which might indicate that the infection was present at least 1 year before the first seizure. Despite enhancement of several cranial nerves, our patient did not have any classic clinical presentations of LNB, such as facial paresis or radiculitis.

The level of the B-cell-attracting chemokine CXCL13 has been found to be elevated in CSF in early LNB, even before *B. burgdorferi* antibodies are present [46, 47]. CXCL13 levels fall rapidly after the start of antibiotic therapy [7, 48]. The CXCL13 concentrations in CSF samples of untreated LNB patients have been found to be significantly higher than the concentrations in the non-LNB group, in the viral central nervous system (CNS) infection samples or samples from patients with non-infectious neuroinflammatory conditions. Thus, CSF CXCL13 appears to be an excellent biomarker for differentiating LNB from viral CNS infections and from other neuroinflammatory conditions when locally determined cut-offs are used. It might also be helpful for monitoring response to antibiotic treatment [7]. Other CNS-prone spirochetes like *Treponema pallidum* induce similar rises in CXCL13 levels in CSF [49, 50]. The CXCL13 concentration in CSF is elevated in CNS lymphoma patients also [51, 52]. Nothing is known so far about CXCL13 levels after neurosurgery. Whether the increase in CSF CXCL13 levels in our patient was due to the ligation of the aneurysm of the left anterior cerebral artery or due to a new episode of LNB remains unknown.

This case report describes an uncommon complication of neuroborreliosis in a child. Multiple aneurysms developed in the 2 years after the meningoencephalitis due to *B. burgdorferi* and manifested as a stroke. Our patient recovered well, but the outcome could have been more severe. This case report underlines the importance of CSF analysis and diagnosis of a potential CNS infection in paediatric patients with neurological symptoms.
